# Glances at the Hospitals

**Published:** 1900-06-30

**Authors:** 


### GLANCES AT THE HOSPITALS.

### BRISTOL GENERAL HOSPITAL.

At the end of 1898 this infirmary had 172 patients in residence, and at the close of 1899 there were only 159. During the year 2,195 patients were admitted. Of these 1,260 were discharged cured, 391 relieved, 30 not improved, 79 by patients' desire, and 186 died. In the out-patients' department the total number of patients treated during 1899 exceeded 33,000. This number shows that there were 1,500 more out-patients than in 1898, and that where other infirmaries are able to reduce these cases the Bristol Hospital increased them. At the Bristol Eye Hospital 5,703 cases were treated; at the Children's Hospital 4,422, and at the Royal Infirmary 32,843. These figures show that nearly one in three of the inhabitants of Bristol are receiving gratuitous medical advice, and in that calculation the Union Infirmary is omitted. It should, however, be stated that the workpeople contributed over £3,000 to the two largest infirmaries. The Jubilee Home was opened was opened by the Queen on November 15th, and the managers say that it is already evident that the home will be the means of considerably increasing the utility of the hospital, as it will permit the admission of fresh cases to the beds vacated by the patients transferred to the home. The domestic offices have been improved at a cost of £1,500, and a bacteriological laboratory has been been put up. The out-patients' department is to be altered and enlarged at an estimated expense of £5,000. A very sensible arrangement has been made with the committee of the Bristol Royal Infirmary whereby medical students can see the practice of both institutions.

### THE LEICESTER INFIRMARY.

The annual report for 1899 is the 127th of the existence of the institution. It shows that there were 134 patients in residence at the beginning of the year, and 144 at the end of it. During the year 1,384 patients were admitted by recommendation, 338 as accidents, and 442 as emergencies, making a total of 2,298 in-patients. Of these 1,561 were discharged cured or relieved, 46 at own request, 34 unrelieved, 329 were made out-patients, 137 died, 28 were sent to convalescent homes, and 19 were discharged for various other reasons. The children's hospital had a total of 486 under treatment; and the fever house a total of 107. The average cost of the in-patients was almost £1 1s. per week. Coming to the out-patients' department, we have to chronicle a total of 17,451, making a grand total of 20,342. From the house surgeon's report we learn that 862 major operations and 806 minor were performed during the year. This table would be immensely improved by a column showing the results of these operations, or at least of the major operations. We notice that 146 abdominal sections were performed, and it would have been most interesting to learn how many were successful. So with the 43 excisions, the 36 amputations, the 51 hernias, the 10 nephrotomy cases. All may have recovered, or all may have died for anything the table tells us. Anæsthetics were administered in 1,755 instances. The infirmary might be in a better financial position. The total income was £11,932, and the total expenditure was £13,948. The extra expenditure was caused by the large increase in the number of patients, by the cost of structural improvements in the building, and by "severe and protracted illnesses" amongst the staff. As regards the increase of patients, it would seem that the number of out-patients is double what it was twenty years ago, and that in 1899 alone the increase was 527. Of all departments of an infirmary the out-patients' is that which is most frequently abused. We do not say that it is the case in Leicester, but we do think that it should be carefully watched, and a wage limit established above which free treatment should not be given. We are glad to notice that the workpeople's own donations amounted to £3,600.

### THE KINGSTON-UPON-THAMES VICTORIA HOSPITAL.

This was opened on January 18th, 1899, and since that date to December 31st last 178 patients have been admitted. Of the total number of admissions 66 were medical cases, 74 were surgical, and 38 were accidents. Of the medical cases 8 died, and of the accidents 4 died, while all the surgical cases except 3 were either cured (55) or benefited (16). Judging from the capital account, the hospital would seem to have cost £4,278. It is already too small for the population of the neighbourhood, but funds are not at present forthcoming for its enlargement. Payments by patients in the hospital amounted only to £79 19s., a sum which seems very small for the number of patients treated, and which might surely be considerably increased in the current year. It would be most desirable to state in the next report the amount subscribed by the working classes themselves in aid of this hospital. There are 13 medical men on the honorary staff.

### THE BIRMINGHAM GENERAL HOSPITAL.

During the year 1899 the in-patients reached the respectable total of 4,864, and the out-patients numbered 60,607. These figures show that the in-patients decreased by 49, and that the out-patients increased by 479. The out-patients' department of every hospital ought to be most carefully looked after, as the tendency is always, or nearly always, towards an increase. Adding the number of cases treated at the other charitable institutions in Birmingham, we reach a total of 157,000, without including workhouse infirmary cases. The ordinary income of the Birmingham Hospital for 1899 was £14,133, being about £900 lower than it was for the previous year. The extraordinary income reached £3,100. The managers state that £23,000 a year will be required to keep the hospital up as at present arranged, and at the present moment there is an adverse balance of £7,780. That this hospital does a very large amount of work is proved by the fact that it has a larger average proportion of in-patients to each bed occupied than any other general hospital in the kingdom. We are pleased to note that a systematic inquiry is made into the social circumstances of patients applying for treatment, and that a number "of cases have been referred to their private medical attendant." In some hospitals a wages list is kept, and no one earning above a certain sum can obtain gratuitous treatment. The prevalence in our large towns of a preventible disease like typhoid fever is driven home to us by this report, for we find it stated that it has been necessary to rule that not more than one-third of the beds on the medical side of the house shall be occupied by these fever cases. There are 22 physicians and surgeons on the honorary medical and surgical staff.

### THE BRIDGEND COTTAGE HOSPITAL.

This hospital contained 2 patients at the beginning of the year. There were 61 admissions, 56 were discharged cured or relieved, and 2 died, leaving 5 under treatment on December 31st. The average residence of each patient was 32 days, and the cost per patient per week was 22s. 9d. Although the weekly cost of maintenance is lower than it was in 1898, the increased number of patients treated has caused an adverse balance of £61, and the committee have been compelled to place some restrictions on the number of cases admitted. Mr. Randall has given a donation of £40 to aid in wiping off this debt, so that the amount now wanted is not large, and will, doubtless, soon be forthcoming; but what is essential to the future working of the hospital is a substantial increase in the annual subscriptions.

